# Supplementary material for: Microbial communities living inside plant leaves or on the leaf surface are differently shaped by environmental cues
Source: ISME Commun. 2024 Aug 8;4(1):ycae103. doi: 10.1093/ismeco/ycae103 (PMC11333920; doi:10.1093/ismeco/ycae103)
Supplement: Supplementary_Figures_ycae103 [file supplementary_figures_ycae103.pdf]

Supplementary figures

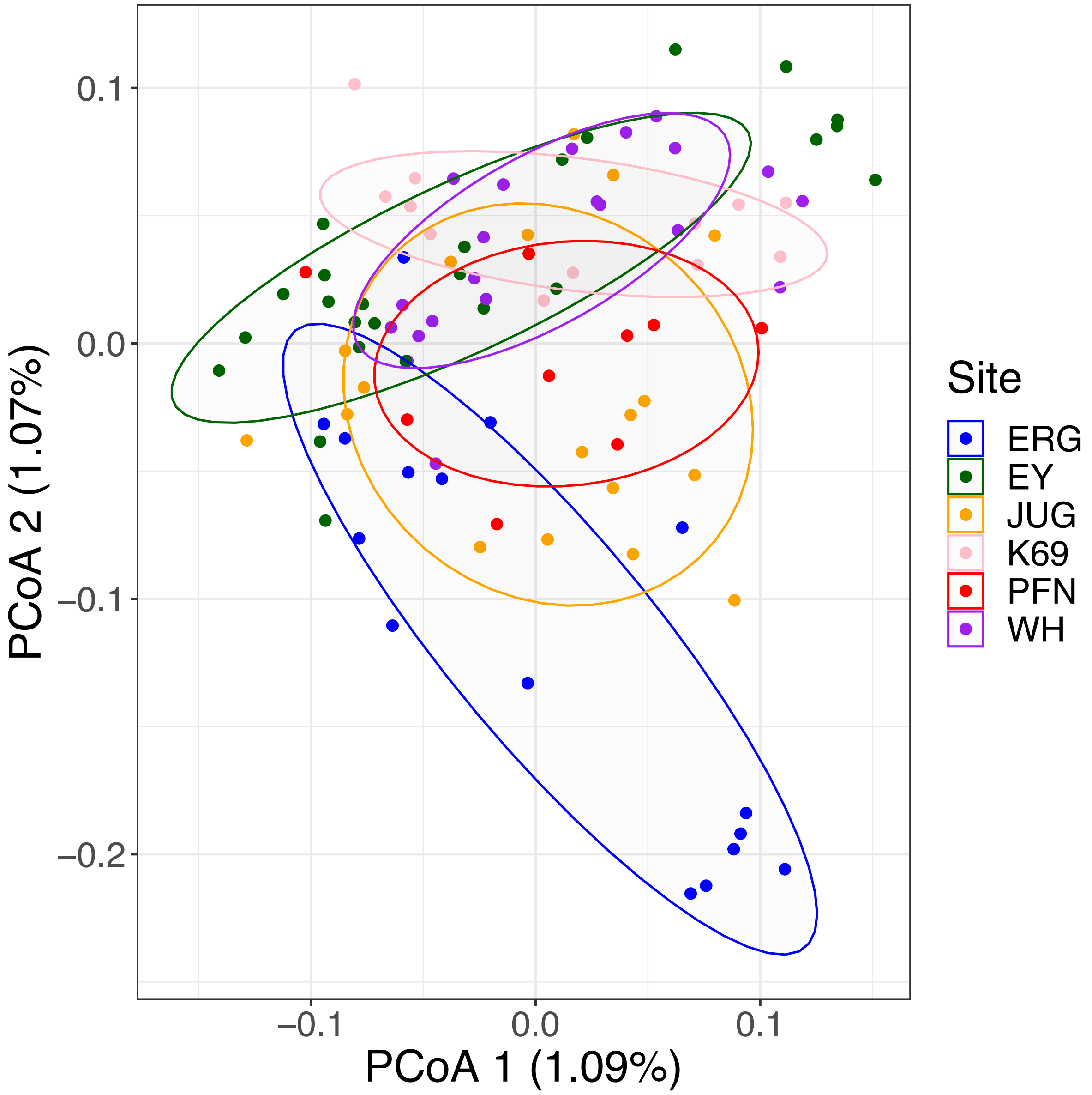

Supplementary figure 1. Genetic variation of the host among sites. Principal coordinate analysis (PCoA) was conducted on single nucleotide polymorphisms (SNPs) using Bray-Curtis dissimilarities. Points represent individual samples, with clusters indicating distinct sampling sites.

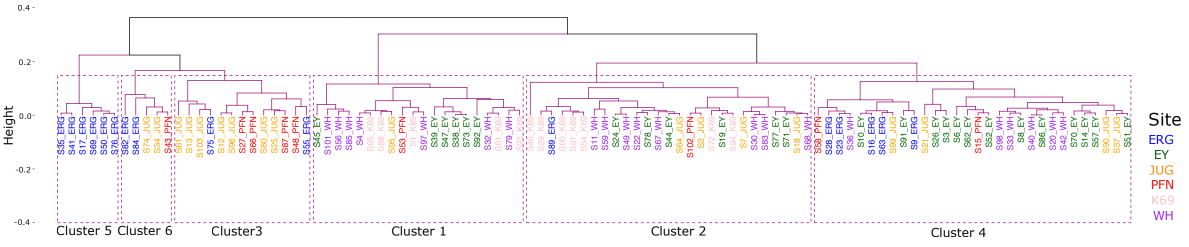

Supplementary Figure 2. Clustering of host genotypes based on SNPs. The dendrogram illustrates the distances between samples based on the SNP table (measured by Bray-Curtis

dissimilarities). Six genotype clusters were identified, with nodes (labeled as sample number and sampling site) colored according to sampling sites.

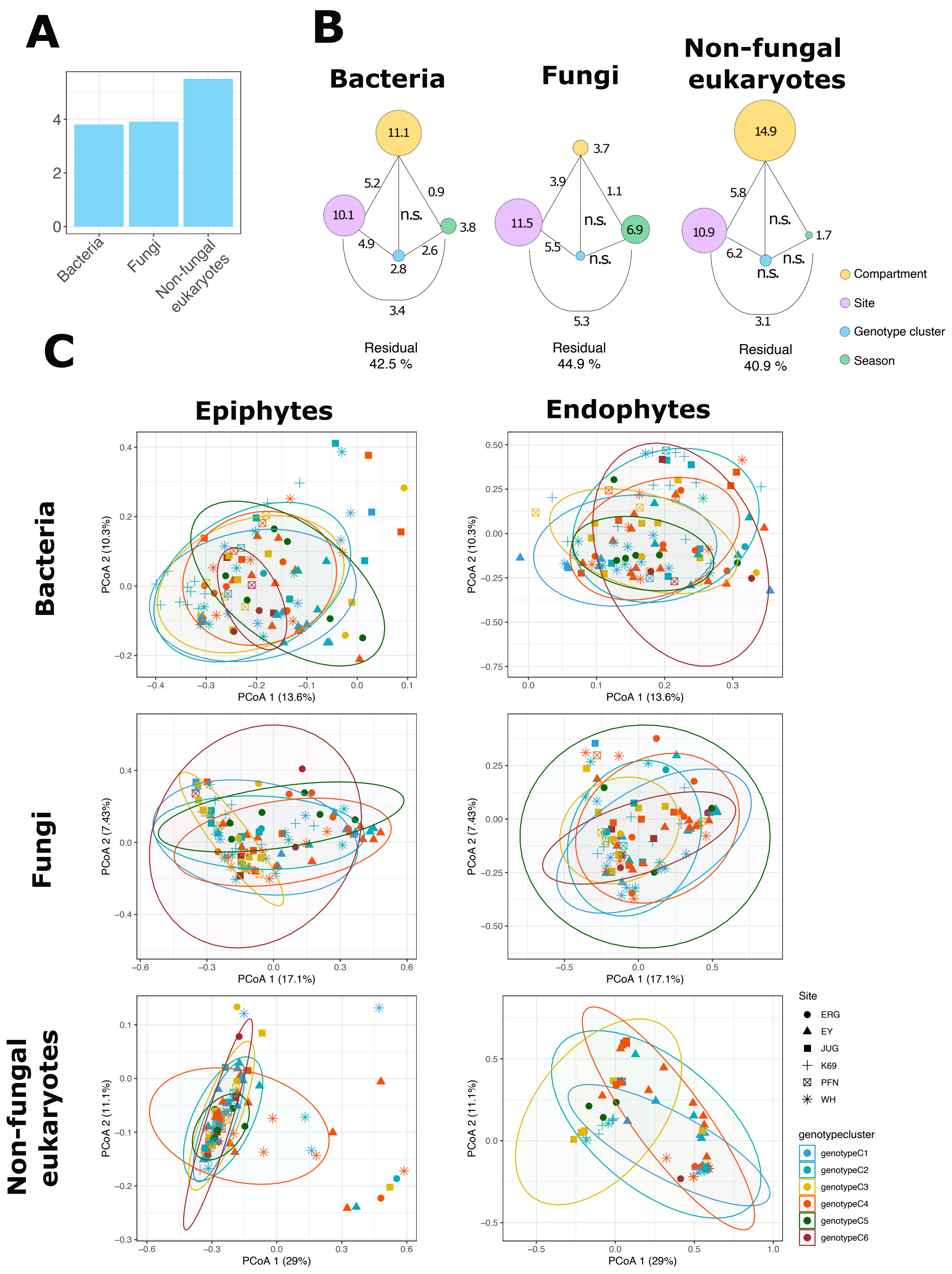

Supplementary Figure 3. Multivariate analysis on genetic variation of the host in structuring leaf microbial communities. (A) A PERMANOVA analysis on Bray-Curtis dissimilarities shows host genotypes' effects on microbiome variation. Bars indicate the percentage of variance explained by host genotype. **(B)** A PERMANOVA analysis shows the

host genotype’s effects on microbial variation in interaction with other factors. Circles depict the percentage of variance explained by the factors ’compartment’, ’site’, ’genotype’, and ’season’; connecting lines depict the percentage of variance explained by interactions between these factors. Only significant effects are shown (permutations = 10,000, $P \leq 0.05$, explanatory categorical variables: compartment x site x genotype x season). **(C)** Principal coordinate analysis (PCoA) of host genotype among sampling sites in epiphytic and endophytic compartments in bacterial, fungal, and non-fungal eukaryotes.

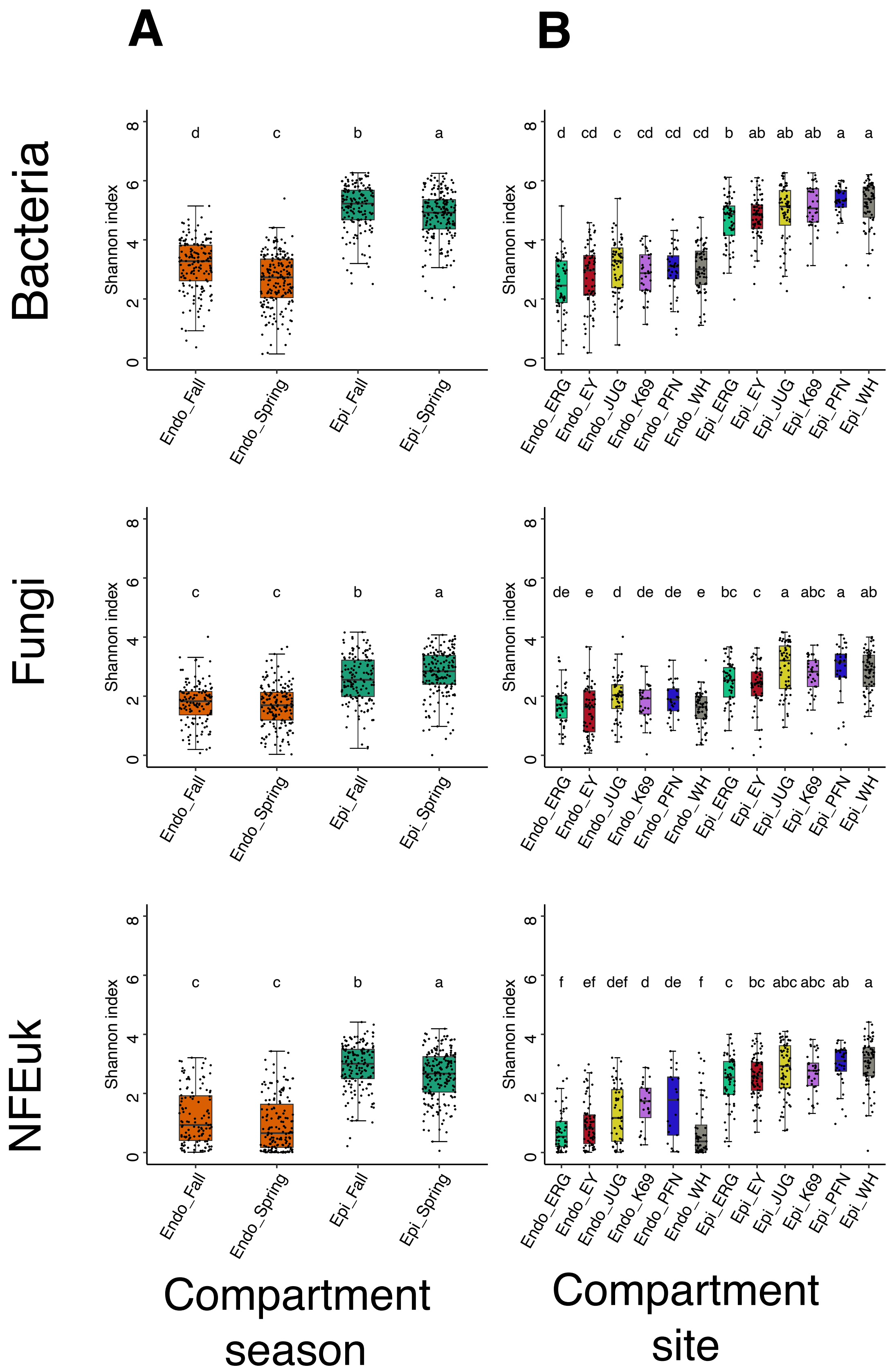

Supplementary figure 4. Within-sample diversity of leaf compartments across sampling sites and sampling seasons. Alpha diversity of epiphytic and endophytic samples across spring and fall samples **(A)** and sampling sites **(B)** in bacteria, fungi, and non-fungal eukaryotic

communities. The box plots display individual samples as dots. Different letters indicate significant group differences (Dunn test, $P < 0.05$).

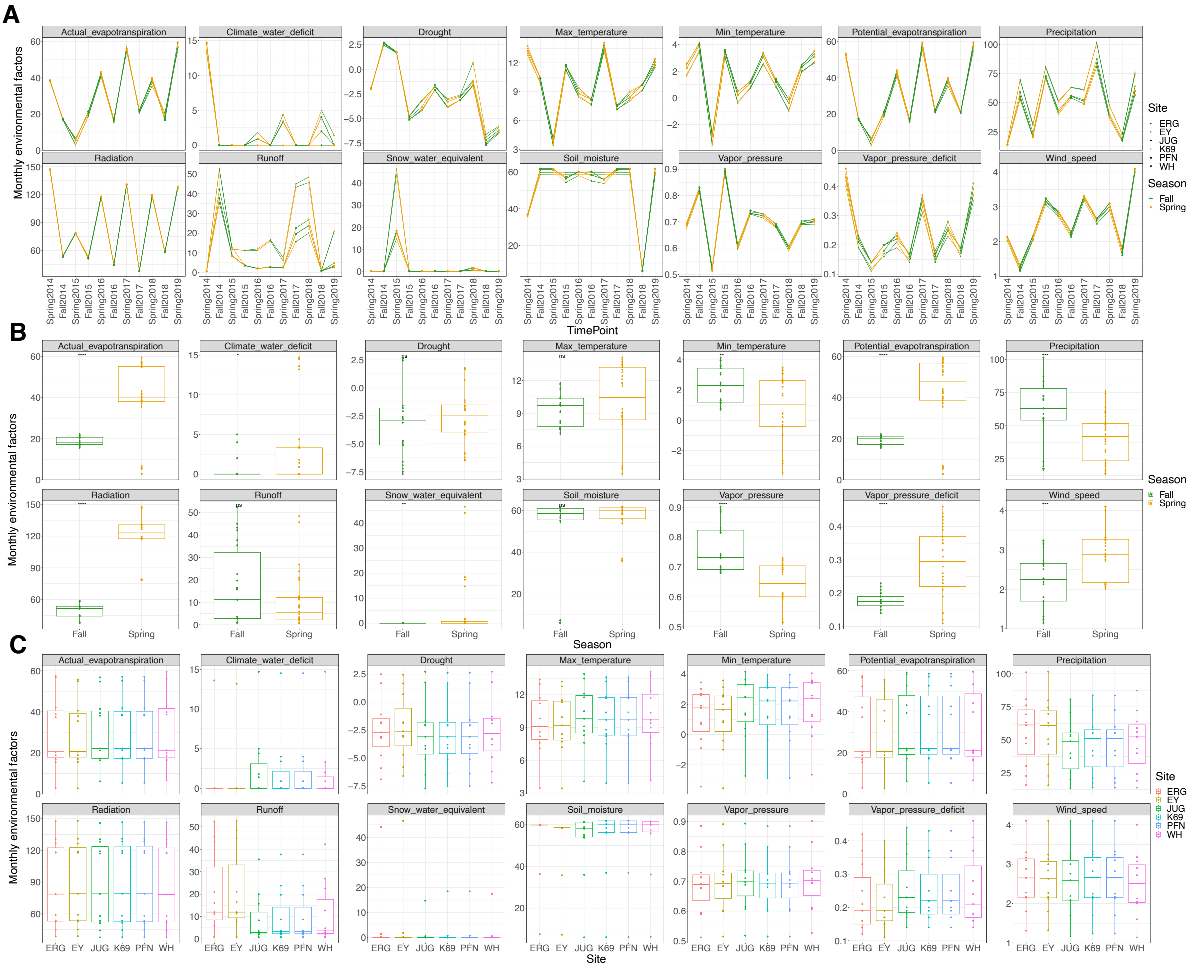

Supplementary figure 5. Changes in environmental variables over sampling time points and sampling sites. **(A)** Lines show the average values for each environmental factor for each sampling month and site. **(B)** Spring vs fall differences for each environmental factor **(C)** Differences between sites for each environmental factor. Asterisks indicate significant differences based on Wilcoxon's test: n.s. ($P > 0.05$), * ($P \leq 0.05$), ** ($P \leq 0.01$), *** ($P \leq 0.001$), and **** ($P \leq 0.0001$).

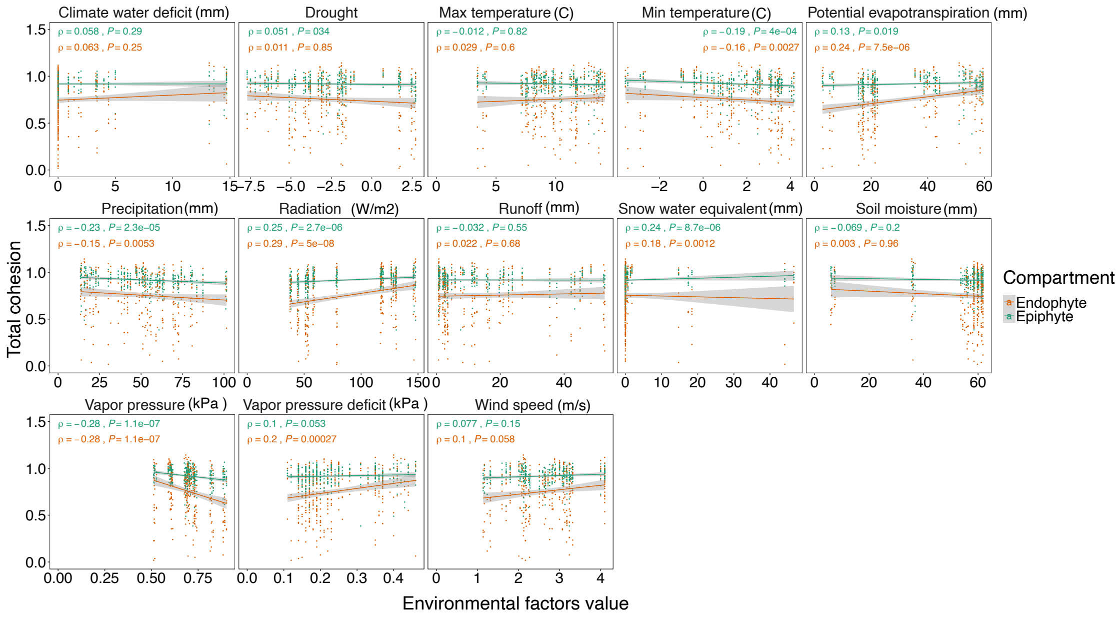

Supplementary figure 6. Relationship between total cohesion and environmental factors. Each plot shows a linear regression model fit to the data to show the association of total cohesion and environmental factors, across compartments. Samples are represented by dots and colored by compartments. The grey lines indicate 95% confidence intervals, and the Spearman correlation coefficient ρ and actual P-values are shown.
