# Supplementary material for: Microbial communities living inside plant leaves or on the leaf surface are differently shaped by environmental cues
Source: ISME Commun. 2024 Aug 8;4(1):ycae103. doi: 10.1093/ismeco/ycae103 (PMC11333920; doi:10.1093/ismeco/ycae103)
Supplement: TableS6_Primers_ycae103 [file tables6_primers_ycae103.docx]

**Supplementary Table 6.** Primers and blocking oligos used in this study.

| Types | Targets | Primer name | Primer sequence (5’-to-3’ orientation) |
| --- | --- | --- | --- |
| Amplification primers | Bacteria 16S rRNA | 799F | AACMGGATTAGATACCCKG |
| Amplification primers | Bacteria 16S rRNA | 1192R | ACGTCATCCCCACCTTCC |
| Amplification primers | Fungi ITS2 | fITS7 | GTGARTCATCGAATCTTTG |
| Amplification primers | Fungi ITS2 | ITS4 | TCCTCCGCTTATTGATATGC |
| Amplification primers | Eukaryotes 18S rRNA | F1422 | ATAACAGGTCTGTGATGCCC |
| Amplification primers | Eukaryotes 18S rRNA | R1797 | TGATCCTTCTGCAGGTTCACCTAC |
| blocking oligos | A.thaliana Mitochondria | 5M30-F | AGATCAGGGGCTCAGCTAACGCGTGAACAC |
| blocking oligos | A.thaliana Mitochondria | cl1BV5-R | TTTTGGCAGGGCGTACTAAACCCACTTACT |
| blocking oligos | A.thaliana ITS2 | cl1ITS2-F | CGTCTGCCTGGGTGTCACAAATCGTCGTCC |
| blocking oligos | A.thaliana ITS2 | clITS2-R | CCTGGTGTCGCTATATGGACTTTGGGTCAT |
| blocking oligos | A.thaliana 18S rRNA | Gc006 | TGATGTATTCAACGAGTTCACACCTTGGCCGACAG |
| blocking oligos | A.thaliana 18S rRNA | Gc008 | TCTAAATGATAAGGTTTAGTGGACTTCTCGCGACG |
| blocking oligos | Albugo 18S rRNA | Gc014 | ATGCGTTCAACGAGTTTAAAACCTTGACCGATAGG |
| blocking oligos | Albugo 18S rRNA | Gc016 | TGGCGAGGTTAAAAAAAGTTCCCACAAATCCATTCGCAAT |

PhiX sequence:

| **>NC_001422.1 Enterobacteria phage phiX174 sensu lato, complete genome**  GAGTTTTATCGCTTCCATGACGCAGAAGTTAACACTTTCGGATATTTCTGATGAGTCGAAAAATTATCTT  GATAAAGCAGGAATTACTACTGCTTGTTTACGAATTAAATCGAAGTGGACTGCTGGCGGAAAATGAGAAA  ATTCGACCTATCCTTGCGCAGCTCGAGAAGCTCTTACTTTGCGACCTTTCGCCATCAACTAACGATTCTG  TCAAAAACTGACGCGTTGGATGAGGAGAAGTGGCTTAATATGCTTGGCACGTTCGTCAAGGACTGGTTTA  GATATGAGTCACATTTTGTTCATGGTAGAGATTCTCTTGTTGACATTTTAAAAGAGCGTGGATTACTATC  TGAGTCCGATGCTGTTCAACCACTAATAGGTAAGAAATCATGAGTCAAGTTACTGAACAATCCGTACGTT  TCCAGACCGCTTTGGCCTCTATTAAGCTCATTCAGGCTTCTGCCGTTTTGGATTTAACCGAAGATGATTT  CGATTTTCTGACGAGTAACAAAGTTTGGATTGCTACTGACCGCTCTCGTGCTCGTCGCTGCGTTGAGGCT  TGCGTTTATGGTACGCTGGACTTTGTGGGATACCCTCGCTTTCCTGCTCCTGTTGAGTTTATTGCTGCCG  TCATTGCTTATTATGTTCATCCCGTCAACATTCAAACGGCCTGTCTCATCATGGAAGGCGCTGAATTTAC  GGAAAACATTATTAATGGCGTCGAGCGTCCGGTTAAAGCCGCTGAATTGTTCGCGTTTACCTTGCGTGTA  CGCGCAGGAAACACTGACGTTCTTACTGACGCAGAAGAAAACGTGCGTCAAAAATTACGTGCGGAAGGAG  TGATGTAATGTCTAAAGGTAAAAAACGTTCTGGCGCTCGCCCTGGTCGTCCGCAGCCGTTGCGAGGTACT  AAAGGCAAGCGTAAAGGCGCTCGTCTTTGGTATGTAGGTGGTCAACAATTTTAATTGCAGGGGCTTCGGC  CCCTTACTTGAGGATAAATTATGTCTAATATTCAAACTGGCGCCGAGCGTATGCCGCATGACCTTTCCCA  TCTTGGCTTCCTTGCTGGTCAGATTGGTCGTCTTATTACCATTTCAACTACTCCGGTTATCGCTGGCGAC  TCCTTCGAGATGGACGCCGTTGGCGCTCTCCGTCTTTCTCCATTGCGTCGTGGCCTTGCTATTGACTCTA  CTGTAGACATTTTTACTTTTTATGTCCCTCATCGTCACGTTTATGGTGAACAGTGGATTAAGTTCATGAA  GGATGGTGTTAATGCCACTCCTCTCCCGACTGTTAACACTACTGGTTATATTGACCATGCCGCTTTTCTT  GGCACGATTAACCCTGATACCAATAAAATCCCTAAGCATTTGTTTCAGGGTTATTTGAATATCTATAACA  ACTATTTTAAAGCGCCGTGGATGCCTGACCGTACCGAGGCTAACCCTAATGAGCTTAATCAAGATGATGC  TCGTTATGGTTTCCGTTGCTGCCATCTCAAAAACATTTGGACTGCTCCGCTTCCTCCTGAGACTGAGCTT  TCTCGCCAAATGACGACTTCTACCACATCTATTGACATTATGGGTCTGCAAGCTGCTTATGCTAATTTGC  ATACTGACCAAGAACGTGATTACTTCATGCAGCGTTACCATGATGTTATTTCTTCATTTGGAGGTAAAAC  CTCTTATGACGCTGACAACCGTCCTTTACTTGTCATGCGCTCTAATCTCTGGGCATCTGGCTATGATGTT  GATGGAACTGACCAAACGTCGTTAGGCCAGTTTTCTGGTCGTGTTCAACAGACCTATAAACATTCTGTGC  CGCGTTTCTTTGTTCCTGAGCATGGCACTATGTTTACTCTTGCGCTTGTTCGTTTTCCGCCTACTGCGAC  TAAAGAGATTCAGTACCTTAACGCTAAAGGTGCTTTGACTTATACCGATATTGCTGGCGACCCTGTTTTG  TATGGCAACTTGCCGCCGCGTGAAATTTCTATGAAGGATGTTTTCCGTTCTGGTGATTCGTCTAAGAAGT  TTAAGATTGCTGAGGGTCAGTGGTATCGTTATGCGCCTTCGTATGTTTCTCCTGCTTATCACCTTCTTGA  AGGCTTCCCATTCATTCAGGAACCGCCTTCTGGTGATTTGCAAGAACGCGTACTTATTCGCCACCATGAT  TATGACCAGTGTTTCCAGTCCGTTCAGTTGTTGCAGTGGAATAGTCAGGTTAAATTTAATGTGACCGTTT  ATCGCAATCTGCCGACCACTCGCGATTCAATCATGACTTCGTGATAAAAGATTGAGTGTGAGGTTATAAC  GCCGAAGCGGTAAAAATTTTAATTTTTGCCGCTGAGGGGTTGACCAAGCGAAGCGCGGTAGGTTTTCTGC  TTAGGAGTTTAATCATGTTTCAGACTTTTATTTCTCGCCATAATTCAAACTTTTTTTCTGATAAGCTGGT  TCTCACTTCTGTTACTCCAGCTTCTTCGGCACCTGTTTTACAGACACCTAAAGCTACATCGTCAACGTTA  TATTTTGATAGTTTGACGGTTAATGCTGGTAATGGTGGTTTTCTTCATTGCATTCAGATGGATACATCTG  TCAACGCCGCTAATCAGGTTGTTTCTGTTGGTGCTGATATTGCTTTTGATGCCGACCCTAAATTTTTTGC  CTGTTTGGTTCGCTTTGAGTCTTCTTCGGTTCCGACTACCCTCCCGACTGCCTATGATGTTTATCCTTTG  AATGGTCGCCATGATGGTGGTTATTATACCGTCAAGGACTGTGTGACTATTGACGTCCTTCCCCGTACGC  CGGGCAATAACGTTTATGTTGGTTTCATGGTTTGGTCTAACTTTACCGCTACTAAATGCCGCGGATTGGT  TTCGCTGAATCAGGTTATTAAAGAGATTATTTGTCTCCAGCCACTTAAGTGAGGTGATTTATGTTTGGTG  CTATTGCTGGCGGTATTGCTTCTGCTCTTGCTGGTGGCGCCATGTCTAAATTGTTTGGAGGCGGTCAAAA  AGCCGCCTCCGGTGGCATTCAAGGTGATGTGCTTGCTACCGATAACAATACTGTAGGCATGGGTGATGCT  GGTATTAAATCTGCCATTCAAGGCTCTAATGTTCCTAACCCTGATGAGGCCGCCCCTAGTTTTGTTTCTG  GTGCTATGGCTAAAGCTGGTAAAGGACTTCTTGAAGGTACGTTGCAGGCTGGCACTTCTGCCGTTTCTGA  TAAGTTGCTTGATTTGGTTGGACTTGGTGGCAAGTCTGCCGCTGATAAAGGAAAGGATACTCGTGATTAT  CTTGCTGCTGCATTTCCTGAGCTTAATGCTTGGGAGCGTGCTGGTGCTGATGCTTCCTCTGCTGGTATGG  TTGACGCCGGATTTGAGAATCAAAAAGAGCTTACTAAAATGCAACTGGACAATCAGAAAGAGATTGCCGA  GATGCAAAATGAGACTCAAAAAGAGATTGCTGGCATTCAGTCGGCGACTTCACGCCAGAATACGAAAGAC  CAGGTATATGCACAAAATGAGATGCTTGCTTATCAACAGAAGGAGTCTACTGCTCGCGTTGCGTCTATTA  TGGAAAACACCAATCTTTCCAAGCAACAGCAGGTTTCCGAGATTATGCGCCAAATGCTTACTCAAGCTCA  AACGGCTGGTCAGTATTTTACCAATGACCAAATCAAAGAAATGACTCGCAAGGTTAGTGCTGAGGTTGAC  TTAGTTCATCAGCAAACGCAGAATCAGCGGTATGGCTCTTCTCATATTGGCGCTACTGCAAAGGATATTT  CTAATGTCGTCACTGATGCTGCTTCTGGTGTGGTTGATATTTTTCATGGTATTGATAAAGCTGTTGCCGA  TACTTGGAACAATTTCTGGAAAGACGGTAAAGCTGATGGTATTGGCTCTAATTTGTCTAGGAAATAACCG  TCAGGATTGACACCCTCCCAATTGTATGTTTTCATGCCTCCAAATCTTGGAGGCTTTTTTATGGTTCGTT  CTTATTACCCTTCTGAATGTCACGCTGATTATTTTGACTTTGAGCGTATCGAGGCTCTTAAACCTGCTAT  TGAGGCTTGTGGCATTTCTACTCTTTCTCAATCCCCAATGCTTGGCTTCCATAAGCAGATGGATAACCGC  ATCAAGCTCTTGGAAGAGATTCTGTCTTTTCGTATGCAGGGCGTTGAGTTCGATAATGGTGATATGTATG  TTGACGGCCATAAGGCTGCTTCTGACGTTCGTGATGAGTTTGTATCTGTTACTGAGAAGTTAATGGATGA  ATTGGCACAATGCTACAATGTGCTCCCCCAACTTGATATTAATAACACTATAGACCACCGCCCCGAAGGG  GACGAAAAATGGTTTTTAGAGAACGAGAAGACGGTTACGCAGTTTTGCCGCAAGCTGGCTGCTGAACGCC  CTCTTAAGGATATTCGCGATGAGTATAATTACCCCAAAAAGAAAGGTATTAAGGATGAGTGTTCAAGATT  GCTGGAGGCCTCCACTATGAAATCGCGTAGAGGCTTTGCTATTCAGCGTTTGATGAATGCAATGCGACAG  GCTCATGCTGATGGTTGGTTTATCGTTTTTGACACTCTCACGTTGGCTGACGACCGATTAGAGGCGTTTT  ATGATAATCCCAATGCTTTGCGTGACTATTTTCGTGATATTGGTCGTATGGTTCTTGCTGCCGAGGGTCG  CAAGGCTAATGATTCACACGCCGACTGCTATCAGTATTTTTGTGTGCCTGAGTATGGTACAGCTAATGGC  CGTCTTCATTTCCATGCGGTGCACTTTATGCGGACACTTCCTACAGGTAGCGTTGACCCTAATTTTGGTC  GTCGGGTACGCAATCGCCGCCAGTTAAATAGCTTGCAAAATACGTGGCCTTATGGTTACAGTATGCCCAT  CGCAGTTCGCTACACGCAGGACGCTTTTTCACGTTCTGGTTGGTTGTGGCCTGTTGATGCTAAAGGTGAG  CCGCTTAAAGCTACCAGTTATATGGCTGTTGGTTTCTATGTGGCTAAATACGTTAACAAAAAGTCAGATA  TGGACCTTGCTGCTAAAGGTCTAGGAGCTAAAGAATGGAACAACTCACTAAAAACCAAGCTGTCGCTACT  TCCCAAGAAGCTGTTCAGAATCAGAATGAGCCGCAACTTCGGGATGAAAATGCTCACAATGACAAATCTG  TCCACGGAGTGCTTAATCCAACTTACCAAGCTGGGTTACGACGCGACGCCGTTCAACCAGATATTGAAGC  AGAACGCAAAAAGAGAGATGAGATTGAGGCTGGGAAAAGTTACTGTAGCCGACGTTTTGGCGGCGCAACC  TGTGACGACAAATCTGCTCAAATTTATGCGCGCTTCGATAAAAATGATTGGCGTATCCAACCTGCA |
| --- |
